# Chylous Ascites and Pleural Effusion Treated With Intravenous Octreotide

**DOI:** 10.7759/cureus.8669

**Published:** 2020-06-17

**Authors:** Subash Ghimire, Harshil Shah, Sanjay Paudel, Tsu Jung Yang, Hafiz M Khan

**Affiliations:** 1 Internal Medicine, Guthrie Clinic, Robert Packer Hospital, Sayre, USA; 2 Internal Medicine, Guthrie Clinic, Sayre, USA; 3 Medicine, B.P. Koirala Institute of Health Sciences, Dharan, NPL; 4 Internal Medicine, MultiCare Good Samaritan Hospital, Puyallup, USA; 5 Gastroenterology and Hepatology, Guthrie Clinic, Robert Packer Hospital, Sayre, USA

**Keywords:** chylous ascites, cirrhosis, octreotide

## Abstract

Chylous ascites (CA) is uncommon in cirrhosis. It often presents as diuretic-resistant ascites and is associated with increased mortality. Diagnosis is done by the detection of triglyceride-rich ascitic fluid. There are no published guidelines on the management of CA. We describe the case of a middle-aged female who presented with CA secondary to cirrhosis, and the challenges associated with her treatment and her management with the use of intravenous octreotide.

## Introduction

Chylous ascites (CA) is a rare manifestation of cirrhosis. This has been described in a few cases in the medical literature. It can be congenital as well as acquired [1]. Congenital CA, which is caused by lymphatic dysplasia, presents early in life. Acquired CA is mostly caused by malignancy or trauma. In cirrhosis, CA is associated with high mortality [2]. It commonly manifests as diuretic refractory ascites, and treatment options are limited. The use of octreotide in the management of CA is limited to case reports and series [3]. We describe the case of a 59-year-old-female who presented with CA and was managed with octreotide with a significant reduction in ascitic fluid volume.

## Case presentation

A 59-year-old female with a medical history of alcoholic cirrhosis who had undergone uncomplicated elective splenectomy and distal pancreatectomy for splenic artery aneurysm 2 months ago was admitted to the intensive care unit with acute hypoxic respiratory failure for two weeks. She was found to have increased respiratory rate, diminished breath sounds, and abdominal ascites with shifting dullness. The patient had hypoxia, which was attributed to hepatopulmonary syndrome and compressive atelectasis from hepatic hydrothorax. Common causes of hypoxia, including pneumonia, congestive heart failure, and pulmonary embolism, were excluded during work-up.

The patient underwent diagnostic and therapeutic paracentesis as well as thoracentesis. Both ascitic and pleural fluid chemistries revealed elevated triglyceride levels (268 mg/dL in the ascitic fluid); therefore, a diagnosis of CA was made. Microbiological work-up, including gram stain and cultures, were negative. No malignant or suspicious cells were seen in the cytological analysis. She was started on albumin and diuretic therapy with furosemide and spironolactone for portal hypertension. The patient underwent multiple paracentesis procedures with removal of about 2-3 liters of chylous fluid every 72-96 hours. Due to the recurrence of effusion and need for frequent paracentesis, with poor prognosis, goals of care discussion were planned, and a temporary peritoneal drainage catheter was placed for symptom relief with a plan to discharge the patient on hospice care.

**Figure 1:**
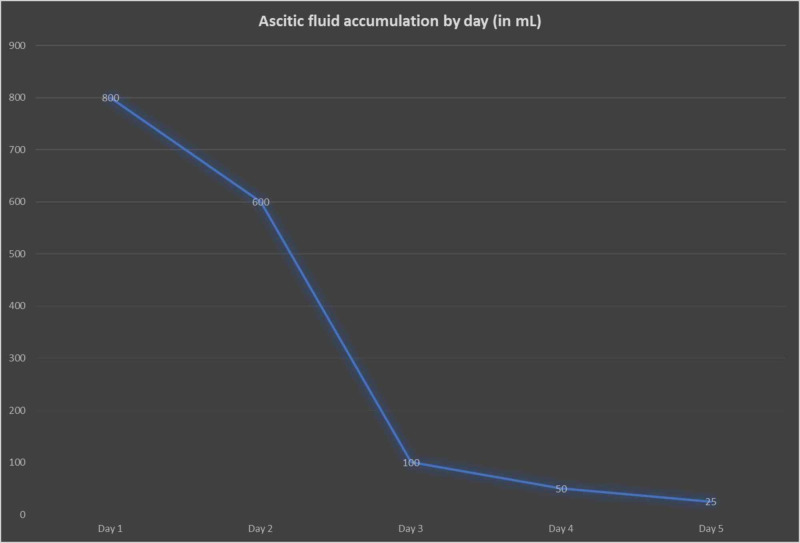
Ascitic fluid volume by day

Despite the above measures, accumulation of ascitic fluid was very challenging to control. She was started on treatment with octreotide 100 micrograms scheduled three times per day. She received it for five days. There was a remarkable improvement in the collection of ascitic fluid during octreotide therapy. The patient’s ascitic fluid collection improved from about 600 mL per day to 25 mL per day, as shown in Figure 1. Her supplemental oxygen requirement improved significantly, and the patient was discharged on diuretic therapy. She underwent TIPSS (transjugular intrahepatic portosystemic shunt surgery) after her clinical improvement. With this, there was a significant improvement in her overall functional status. The patient was referred for liver transplantation.

## Discussion

CA is the collection of milky appearing triglyceride-rich fluid in the peritoneal cavity. It is uncommon and presents challenge in its management. Diagnosis is made when triglyceride level in ascitic fluid is >200 mg/dL [2]. It is important to distinguish CA from pseudo-CA, in which the turbid appearance is due to cellular degeneration from an infection or underlying malignancy without actually containing high level of triglycerides [4]. Abdominal malignancy and cirrhosis account for the majority of cases in developed countries. Infections such as tuberculosis and filariasis are common causes in developing countries [5].

CA is a rare complication of cirrhosis, occurring spontaneously in 0.5-1% of patients. Mechanism of CA in cirrhosis is poorly understood. This can occur as an early presentation or at later stages with cirrhosis or with complications from hepatocellular carcinoma or during TIPSS or procedures related to complications from cirrhosis such as sclerotherapy-related thoracic duct injury [4]. Our patient had spontaneous CA. Although the exact mechanism is unclear, it has been suggested that excessive hepatic and gastrointestinal lymph flow due to elevated portal pressure could lead to a breakage of serosal lymphatic channels, leaking chyle into the peritoneum [5].

The management of CA is equally challenging as no definite guidelines have been suggested [6]. Treatment has to be individualized based on the etiology, severity, and the presence of other comorbidities. Conservative approach includes the use of low-fat diet, medium-chain triglyceride (MCT) intake, total parenteral nutrition (TPN), paracentesis, and use of somatostatin analogs. For resistant cases, options include TIPSS and surgical measures such as closure of retroperitoneal shunt and creation of peritoneovenous shunt [4].

In our case report, we have highlighted the use of octreotide in the management of CA and pleural effusion secondary to cirrhosis. It is also important to note that in our patient, CA developed two months after abdominal surgery; therefore, it was likely not due to trauma from surgery. Somatostatin plays a crucial role in decreasing portal pressure by inhibiting glucagon and other intestinal peptide-mediated splanchnic vasodilation [6]. It also decreases intestinal peristalsis, fat absorption, thoracic duct flow, and triglyceride contents in lymphatics [2]. Octreotide is a somatostatin analog with similar functions but longer half-life (upto two hours), making it advantageous to use through the subcutaneous route [7]. In our patient, we observed significant improvement in the volume of chylous drainage during the five days of treatment with octreotide. The volume of drainage decreased from 600mL/day to 25mL/day. This led to the resolution of symptoms, and the patient was ultimately discharged for further long-term management with TIPSS, which improved her overall functional status.

The successful use of octreotide in the management of CA has been reported in a few other studies. Zhou et al, published results of treatment of six patients with octreotide in 2008, where they reported a significant decrease in cirrhosis-related CA [3]. Berzigotti et al. described the long-term use of octreotide in a cirrhotic patient with portal hypertension. They concluded that there was reduction in the need for repeated paracentesis, with maximum effect seen in the first month and six months with subsequent improvement in quality of life [8]. The long-term use of TPN is believed to have repairing effect on previously ruptured lymph vessels. The successful treatment of iatrogenic chyloperitoneum in a child with octreotide in combination with TPN and MCT has been described in a study by Bhatia et al. [9]. The beneficial role of octreotide has also been described in the management of ascites in the setting of non-cirrhotic portal vein thrombosis, chylous effusion of malignancy origin, and chronic dialysis [6,9,10].

All the aforementioned studies suggest that octreotide along with conservative therapy can play a crucial role in the management of CA and effusion of both cirrhotic and non-cirrhotic origin. Our case showed that even short-term use of octreotide can have a dramatic effect on the management of cirrhosis-related CA and effusion. It not only shortened the hospital stay but also avoided further unnecessary diagnostic tests [11].

## Conclusions

CA is a challenging diagnosis in cirrhotic patients. The use of octreotide can effectively improve chylous fluid accumulation, which provides time for consideration of other treatment measures including TIPSS and liver transplantation.
